# Filamentous prophages in the genomes of *Acinetobacter baumannii* from egypt: impact on biofilm formation and the potential to induce enterotoxicity

**DOI:** 10.1186/s12866-025-04177-z

**Published:** 2025-07-23

**Authors:** Samira M. Hamed, Amira Abdel‑Daim, Samer A. Tadros, Mai M. Zafer

**Affiliations:** 1 Department of Microbiology and Immunology, Faculty of Pharmacy, October University for Modern Sciences and Arts (MSA), Giza, Egypt; 2 Department of Biochemistry, Faculty of Pharmacy, October University for Modern Sciences and Arts (MSA), Giza, Egypt; 3 Microbiology and Immunology Department, Faculty of Pharmacy, Ahram Canadian University, 6th of October, Egypt

**Keywords:** Filamentous phage, *Acinetobacter baumannii*, Whole genome sequencing, Global clones, Zonula occludens toxin, Biofilm, Enterotoxicity

## Abstract

Filamentous phages (FPs) have been recently isolated from *Acinetobacter baumannii*. While FPs are known to modulate the virulence of some Gram-negative pathogens, their role in *A. baumannii* has not been fully explored. This study analyzed 18 clinical isolates of *A. baumannii* from global clones (GC), with draft genomes generated by Illumina sequencing. All isolates were screened for filamentous prophage (FPP) genomes using the Zonula occludens toxin (Zot)-coding gene as a marker. Nine out of the 18 isolates were found to carry *zot* genes. The complete sequences of four FPPs were predicted. FPPs were exclusively found within GC1, GC7, and GC9 strains. Among the *A. baumannii* genomes deposited in the NCBI genomic database, FPPs were found to be disseminated in 42 Pasteur STs spanning at least six GCs, most commonly GC1. The impact of FPs on biofilm formation in *A. baumannii* was investigated using crystal violet assay. None of the *zot*-negative isolates formed strong biofilms, while six (66.6%) *zot*-positive isolates did. The biofilm indices of *zot*-positive isolates were significantly higher compared to *zot*-negative isolates. The potential enterotoxicity of the *zot*-positive strains was also assessed using in silico and experimental methods. The cytotoxic effect of cell-free supernatants (CFSs) on Caco-2 cells was measured by the MTT assay. Cells treated with CFSs from *zot*-positive strains exhibited significantly higher cytotoxicity than those treated with CFSs from *zot*-negative strains. Upon injecting the CFS of a *zot*-positive strain intraperitoneally into BALB/c mice, severe diarrhea was observed within 6 h. Histological examination of the intestinal tissue 24 h post-injection revealed significant changes. In conclusion, this study suggests that FPPs are widely disseminated in *A. baumannii* GCs and may enhance biofilm formation and enterotoxicity, potentially contributing to the pathogen’s virulence.

**Supplementary Information:**

The online version contains supplementary material available at 10.1186/s12866-025-04177-z.

## Introduction

Filamentous phages (FPs) are a group of phages that belong to the family *Inoviridae* [1]. They have a unique genome organization, morphology, and life cycle. With only a few exceptions, FPs are hosted mainly by Gram-negative bacterial genera such as *Escherichia*, *Neisseria*, *Pseudoalteromonas*, *Pseudomonas*, *Ralstonia*, *Salmonella*, *Shewanella*, *Stenotrophomonas*, *Xanthomonas*, and *Yersinia* [1, 2]. Structurally, FPs have circular single-stranded DNA (ssDNA) coding for approximately ten genes [3]. This is packaged into long, flexible filaments made up of thousands of helically arranged subunits of the major coat protein (pVIII). The ends of the filament are fitted with two different proteins. The leading terminus is capped by pVII and pIX proteins, while pIII and pVI cap the terminal end [2]. FPs are sometimes even longer than the bacteria they harbor, ranging in length from 800 to 4000 nanometers [2]. They replicate by integration into the host chromosome, sometimes as tandem repeats [4], or otherwise extra-chromosomally (episomally) in circular, double-stranded plasmid-like replicative forms (RF) [1]. Both forms are capable of producing ssDNA and transcripts. Their replication involves two main steps: minus strand synthesis, where host RNA polymerase synthesizes an RNA primer to initiate conversion of the viral ssDNA into RF double-stranded DNA and plus strand synthesis, which occurs via a rolling-circle mechanism on the RF DNA template [5]. In either case, viral particles are continually shed without causing host cell death [6]. Instead of forming lytic plaques, FP forms opaque zones with reduced growth on bacterial lawns [2].

FPs have been widely reported from diverse bacterial species spanning both clinical and environmental isolates [7]. Rather than harming their bacterial hosts, they were demonstrated to influence their virulence [2]. In *Vibrio cholerae*, cholera toxin (CTX), which is the main virulence factor responsible for cholera, the deadly human disease, is encoded by the FP CTXΦ [8]. Another protein encoded by CTXΦ that contributes to the virulence of *V. cholera* is the zonula occludens toxin (Zot). This protein acts synergistically with the cholera toxin in the small intestine, causing cholera-specific severe dehydrating diarrheal illness [9, 10]. Specifically, Zot promotes the permeability of the small intestine by affecting the structure of the intercellular tight junctions [10]. In addition, Zot plays a crucial role in the assembly and release of the phage virions, FPs were also linked to the strain-specific pathogenicity of the loss of self-transmissibility of Zot mutants [8].

The adhesion and desiccation survival of *Pseudomonas aeruginosa* biofilms were demonstrated to be enhanced by the filamentous phage Pf1, whose genes were also overexpressed in *P. aeruginosa* biofilms [11, 12]. The FP YpfΦ was verified by Derbise et al. (2007) [4] to enhance *Yersinia pestis* multiplication and dissemination capacity in mice. In *Neisseria meningitidis*, the FP MDAΦ was reported to enhance the colonization of the nasopharynx and to be associated with invasive disease [13, 14]. FPs were also linked to the strain-specific pathogenicity of *Escherichia coli*, which causes extraintestinal infections [15]. Renda et al. (2016) [16] described FP in *Acinetobacter baylyi* strain ADP1, which they designated competence-reducing *Acinetobacter* phage (CRAϕ). In their analysis, the authors identified homologous phages in the genomes of some strains of *Acinetobacter baumannii*. They also recommended exploring their potential role in the virulence of this problematic species, a research gap that is yet to be addressed. Recently, Narancic et al. (2024) [17] successfully isolated and characterized FPs from *A. baumannii*. By analyzing 541 complete genomes retrieved from the NCBI database, they identified FPPs in 83 strains, accounting for 15.3% of the total. These FPPs were classified into 10 distinct groups, designated A to J, highlighting their genetic diversity. While FP infection did not notably affect twitching motility or capsule production in *A. baumannii*, it was associated with changes in bacterial growth kinetics, reduced biofilm formation, and increased susceptibility to antibiotics.

Of all FP structural proteins thoroughly reviewed by other authors [2], only the pI protein stands out as being highly conserved throughout the numerous FP lineages. At their N-terminus, they have a conserved Zot domain that is named after the Zot protein, which is the pI homolog found in the *Vibrio* CTXφ phage [2].

Inspired by the existing evidence of the contribution of FPs to the virulence of their host bacteria, and as Zot proteins are conserved in all FPs, the current study aimed at screening the genomes of clinical isolates of *A. baumannii* isolated from Egyptian patients for FPP genomes using Zot-coding genes as markers. To gain more insights into the role of FPs in the virulence of *A. baumannii*, the association of FPs with selected virulence factors, including biofilm formation and enterotoxicity, was also investigated.

## Materials and methods

### Bacterial strains

Eighteen clinical isolates of *A. baumannii* were included in the current study. These were collected in a previous study from patients admitted to Kasr Al-Ainy Hospital during 2020. As part of the previous study, the draft genomes of the isolates were generated by Illumina sequencing [18]. Antimicrobial susceptibility profiles, sequence types (STs), and global clones (GCs) of the isolates were also defined [18].

### Screening *A. baumannii* isolates for *zot* genes

The draft genomes of the 18 isolates were, in silico, screened for genes predicted to encode zona (zonula/zonular) occludens toxin-like proteins using the comparative systems service offered by the Bacterial and Viral Bioinformatics Resource Center (BV-BRC), https://www.bv-brc.org/ [19]. Using the same service, the predicted amino acid sequences of Zot proteins encoded by all strains were extracted for subsequent analysis. Multiple sequence alignment (MSA) of all predicted amino acid sequences of the *zot* genes identified in our isolates was performed using Clustal Omega (https://www.ebi.ac.uk/jdispatcher/msa/clustalo) with default parameters [20]. The aligned sequences were visualized using Jalview version 2.11.2.6 [21].

### Identification of the *zot*-harboring FPPs

For the identification of the *zot*-harboring *A. baumannii* FPPs, the *zot*-positive contigs were explored by SnapGene viewer v5.1.3.1 (from Insightful Science; available at snapgene.com), and their topology was defined by visualizing fastg files generated by SPAdes 3.14.1 [22] in Bandage [23]. While the *zot*-positive contigs were originally annotated in our previous study [18] using the NCBI (National Center for Biotechnology Information) Prokaryotic Genome Annotation Pipeline (PGAP) [24], the predicted amino acid sequences of unannotated genes were analyzed using the BLASTp tool of the NCBI non-redundant protein database (https://blast.ncbi.nlm.nih.gov/Blast.cgi) [25] and InterProScan (https://www.ebi.ac.uk/interpro/search/sequence/) [26] to infer potential protein functions. Circular and linear maps of the FPP genomes and *zot*-positive contigs were generated by the Proksee (https://proksee.ca/) web server [27]. The most similar phage sequences were searched using the nucleotide Basic Local Alignment Search Tool (BLASTn) (https://blast.ncbi.nlm.nih.gov/Blast.cgi) [25] against the NCBI non-redundant nucleotide (nr/nt) database. Trials for the identification of FPPs were done using the PHASTER tool (PHAge Search Tool Enhanced Release), available at: https://phaster.ca/ [28].

### Phylogeny analysis of the FPPs

To explore the closest FPPs to those carried by our isolates, all complete RefSeq genomic sequences of the bacteriophages that belong to the family *Inoviridae* were retrieved from the NCBI genomic database (https://www.ncbi.nlm.nih.gov/datasets/genome/), accessed on April 19, 2025. To minimize redundancy, only one representative genome was included per virus. Together with the FPP sequences identified in our isolates, these were used for creating a proteomic tree using the ViPTree v3.3 server, available at: https://www.genome.jp/viptree/ [29]. The generated proteomic ViPTree was visualized and edited using the interactive Tree of Life v3 (iTOL) tool, available at: https://itol.embl.de/ [30].

In order to determine the phylogenetic placement of the FPPs identified in our isolates among other *A. baumannii* FPs, we followed the classification proposed by Narancic et al. (2024) [17], which is based on the similarity of the amino acid sequence of Zot proteins. A maximum likelihood phylogenetic tree was constructed using MEGA11 [31], incorporating all Zot protein sequences identified in our isolates, along with one representative sequence from each of the ten FP groups (A–J) defined by Narancic et al. (2024) [17].

### Analysis of the ST/GC distribution of FPPs in *A. baumannii* isolates deposited in the NCBI genomic database

To investigate whether FPPs are confined to certain ST/GC (Sequence type/Global Clone) of *A. baumannii*, and following the analysis performed by Narancic et al. (2024) [17], ST/GC distribution of the *zot*-positive *A. baumannii* isolates was investigated. For this purpose, we searched the NCBI nucleotide database (accessed on April 20, 2020) for the nucleotide sequences that encode zonula/zonular occludens toxin-domain containing proteins, limiting our search to the sequences found in *A. baumannii*. Only the isolates with complete genomes were considered for the ST/GC distribution analysis. The STs of all isolates with complete genomes were assigned through the PubMLST server (https://pubmlst.org/abaumannii/) based on the sequence typing schemes of Pasteur [32] and Oxford [33]. As the Oxford ST could not be assigned to some isolates, the Pasteur STs were employed for the GC distribution analysis. For this purpose, a ST^Pas^-based goeBURST analysis was implemented using Phyloviz software [34]. The same software was used for depicting a minimum spanning tree for the allelic profiles of all tested isolates together with all allelic profiles of the Pasteur typing scheme found in the PubMLST database (accessed on April 20, 2025).

### Investigation of the relationship between FPs and biofilm formation

The relationship between carrying *zot* genes and biofilm formation ability was studied by determining and comparing the biofilm index of the *zot*-positive and *zot*-negative isolates. The biofilm index was determined using the crystal violet assay as described before [35]. Briefly, overnight cultures were diluted to 5 × 10^5^ CFU/mL in Luria Bertani (LB) broth. Aliquots of 200 µL were transferred in three biological replicates to a sterile 96-well polystyrene microtiter plate (Greiner Bio-one^®^, Germany) and incubated at 37 °C for 24 h in static conditions. Planktonic cells were then pipetted to another 96-well plate and the turbidity was measured using Stat Fax 2100 Microplate Reader (Awareness Technology Inc., Palm City, FL, USA) at 600 nm (OD_growth_). Wells were then washed three times with sterile phosphate-buffered saline (PBS) to remove loosely adherent cells. After air drying for 30 min, the biofilm was stained with 225 µL 0.1% crystal violet solution for 15 min, washed three times with water then allowed to air dry for one hour. The biofilm-associated dye was then solubilized by 30 % acetic acid in water, which was then quantified at 570 nm (OD_Biofilm_). The biofilm formation capacities of the isolates were expressed as Biofilm Formation Index (BFI) by which the biofilm biomass is normalized to the growth of each isolate. The following equation was used for calculating the BFI of each isolate:

$$\frac{BFI=\left[{OD}_{biofilm}-{OD}_{biofilm\;control}\right]}{\left[{OD}_{growth}-{OD}_{growth\;control}\right]}$$

Based on their BFIs [36], the isolates were allocated into one of four biofilm formation categories including: strong, moderate, weak, and negative.

### Investigation of the association between FPs and enterotoxicity

#### In silico analysis of Zot protein sequences

Our prediction for the enterotoxicity of the FP proteins was mainly based on the structural analysis of Zot proteins.

#### Toxin prediction of Zot proteins

Toxin prediction of *zot* gene products was done using the toxin prediction tool provided by BTXpred server (http://www.imtech.res.in/raghava/btxpred/) as described before [37]. The same analysis was done for *V. cholerae* N16961 Zot (GenBank accession: P38442). With at least 95% accuracy, the tool predicts and classifies toxins into endo- or exotoxins using support vector machines (SVM)-based modules, hidden Markov models (HMM), and PSI-Blast. Besides, the tool identifies the function of the potential toxins with high accuracy [38].

#### Identification of Zot domains and motifs

The similarity between Zot protein sequences produced by *A. baumannii* isolates and those produced by other enteric pathogens, such as *V. cholera* N16961 (GenBank: YP_004286239.1), *Vibrio parahaemolyticus* PMC53.7 (GenBank: OOX72886.1), and *Campylobacter concisus* 13,826 (GenBank: ABV23516.1), was analyzed. For this purpose, multiple sequence alignments were performed by Clustal Omega (https://www.ebi.ac.uk/jdispatcher/msa/clustalo) [20] which was also used to generate the similarity matrix. The analysis was also expanded to include Zot proteins encoded by other human pathogens, such as *P. aeruginosa* PAK (GenBank: NP_039606.1), and *N. meningitidis* MC58 (GenBank: AAF42092.1). The aligned sequences were visualized by Jalview version 2.11.2.6 [21]. The motifs Walker A (GxxxxGK[S/T], where x is any residue) and Walker B (hhhh[D/E], where h is a hydrophobic residue) known to be characteristic of the p-loop NTPases were investigated [39]. In addition, all protein sequences were screened for the Zot receptor binding motif previously proposed for *V. cholera* Zot protein (GXXXVQXG) [9].

#### Experimental analysis of the association between FPs and enterotoxicity

##### Preparation of cell-free supernatants (CFSs)

Bacterial cell-free supernatants (CFSs) were used for cytotoxicity assays. CFSs were prepared from overnight cultures of Brain Heart Infusion broth inoculated by 10^6^ cfu/mL of each strain and incubated at 37º C with shaking at 150 rpm. After centrifugation at 3000 rpm for 15 min, the CFSs were filter-sterilized through 0.22 μm membranes (Millipore, USA) and immediately stored at −20 ℃ before use in cytotoxicity and in vivo assays (M12 only).

##### In vitro cytotoxicity MTT assay

Cytotoxicity test was determined in Caco-2 colorectal cancer cell line (ATCC, Manassas, VA, USA). Cells were grown in Dulbecco's Modified Eagle Medium (DMEM; Gibco™, NE, USA) supplemented with 10% fetal bovine serum (FBS; Gibco™, NY, USA), 100 units/mL of penicillin, and 100 mg/mL of streptomycin. Cells were subcultured to pre-confluence and were maintained at 37 °C in a humidified atmosphere with 5% CO_2_.

The assay was done using the bacterial cell-free supernatants (CFSs) of (1) four bacterial isolates carrying different alleles of the complete *zot* gene (M2, M3, M12, and M18); (2) a bacterial strain that carries an incomplete *zot* gene (M14); and (3) a *zot*-negative bacterial strain (M1) that served as a negative control. All CFSs were diluted in DMEM media at 37 °C to give five concentrations of two-fold serial dilutions. Monolayers of Caco-2 cells were grown in 96-well microtiter plates (10^3^ cells/well) for 24 h. Cells were incubated with various concentrations of the bacterial CFSs. Then, 20 µL of MTT (5 mg/mL) was added to each well and incubated at 37 °C for 4 h. The media was then carefully removed, and 150 µL of MTT solvent was added. The plate was covered with tin foil, and the cells were agitated on an orbital shaker for 15 min. Finally, the OD was measured at 570 nm in a microplate reader (BMG LABTECH^®^ FLUOstar Omega, Germany). The untreated cells represent the control, and the surviving percentage was calculated from three technical replicates by dividing the OD of treated cells by the OD of the control, untreated cells. The 50% inhibitory concentration (IC50) values were obtained from the transformed curves (GraphPad Prism software, Version 9.1.1). The experiment was done at 37 °C in a CO_2_ environment.

Caco-2 cells at a concentration of 3 × 10^5^/well were seeded in a 6-well plate for 24 h. Then, 100 µL of different CFSs were added, and the cells were incubated for 48 h. The morphological changes of Caco-2 cells were examined under an inverted light microscope (Olympus, Tokyo, Japan) after 48 h.

##### In vivo assessment of the enterotoxic activity

The enterotoxic activity of the CFS from the *zot*-positive strain (M12), which demonstrated the highest in vitro cytotoxicity to Caco-2 cells, was tested in vivo using adult male BALB/c mice weighing 27–32 g. Mice used in this study was housed in and obtained from the animal facility at the Faculty of Pharmacy, Ahram Canadian University, Egypt. The study was conducted at the experimental animal facility of ACU and approved by the IRB at the Faculty of Pharmacy, ACU with protocol approval number REC2023. All animal experiments comply with Guide for the care and use of laboratory animals, 8th Edition 2011, by National Research Council (US). The mice were housed at a constant temperature of 24˚C under a 12-hour light/12-hour dark cycle with free access to regular pellet food and water. They were acclimatized to the environment for one week before the start of the experiments. The mice were randomly divided into two groups (*n* = 3). In the first group (control group), each mouse received 0.2 mL of saline intraperitoneally. The second group received 0.2 ml of CFS intraperitoneally. The mice were observed for 24 h and then sacrificed by cervical dislocation under prior anesthesia by IP injection of ketamine/xylazine mixture (90/10 mg/kg respectively). Autopsy samples were taken from the colons of the mice in both groups and fixed in 10% formal saline for 24 h. The samples were washed in tap water, and then dehydrated using serial dilutions of alcohol (methyl, ethyl, and absolute ethyl). The specimens were cleared in xylene and embedded in paraffin at 56˚C in a hot air oven for 24 h. Paraffin beeswax tissue blocks were prepared and sectioned at 4 microns thickness using a rotary LEITZ microtome. The obtained tissue sections were collected on glass slides, deparaffinized, and stained with hematoxylin and eosin stain [40] for examination through the light electric microscope.

### Statistical analysis

The normality of the studied groups was assessed using the Shapiro–Wilk test. As the data were found to follow a Gaussian distribution (P-value > 0.05), parametric tests were applied for comparing group means. Group comparisons of the BFIs and the cytotoxicity assay results were performed using the unpaired t-test for two-group comparisons and one-way ANOVA for comparisons involving more than two groups, as appropriate. P-values < 0.05 were considered statistically significant. All statistical analyses were conducted using GraphPad Prism 8 software (GraphPad Software, USA).

## Results

### Prevalence of *zot* genes and features of the *zot*-positive isolates

Zot-coding genes were identified in the genomes of nine out of eighteen isolates (50.0%). These were exclusively found in three GCs including GC1, GC7, and GC9. Only six isolates carried complete *zot* gene sequences. Two genes were incompletely sequenced, while one gene, carried by M14, was interrupted by an unknown sequence (incomplete in the middle of the contig) and hence considered non-functional. Three isolates carried two copies of the *zot* gene. These include M9, M12, and M18. In M9 and M12, the two *zot* genes were carried on the same contig, while M18 carried two different alleles of the *zot* gene on two different contigs. Together with their metadata, susceptibility profiles, and STs/GCs, the *zot*-positive isolates are shown in Table 1. The predicted amino acid sequences of the protein products of the complete *zot* genes ranged from 354 to 414 aa. Variations in amino acid composition are illustrated in the MSA shown in Supplementary Fig. 1.

**Table 1:** Features of the *zot*-positive isolates

Isolate No.	ST^Pas^	ST^Oxf^	GC	Specimen type	Predicted Zot proteins (size)	Antimicrobial Susceptibility Profiles
M12	19	1604/231	1	Blood	Two complete sequences (414 aa and 391 aa)	XDR
M15				Wound swab	One complete sequence (414 aa)	MDR
M6	ND^*^	ND^*^	ND^*^	Sputum	One partial sequence (298 aa)	MDR
M9				Blood	Two complete sequences (414 aa and 391 aa)	XDR
M2	85	1089	9	Wound swab	One complete sequence (354 aa)	XDR
M11		1089		Pleural fluid	One complete sequence (354 aa)	XDR
M18		1078		Blood	Two complete sequences (388 aa and 354 aa)	XDR
M3	113	2246	7	Blood	One partial sequence (312 aa)	XDR
M14		2329		Urine	One partial sequence (307 aa)	XDR

MDR multidrug-resistant, XDR extensively drug-resistant, ST^Pas^ sequence type based on Pasteur scheme, ST^Oxf^ sequence type based on Oxford scheme

### *zot*-harboring FPPs

**Fig. 1:**
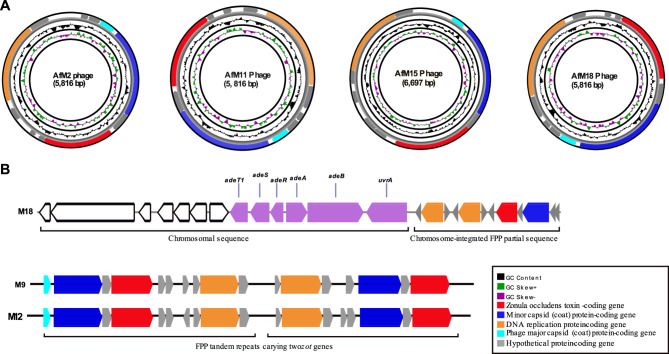
Genetic maps of circular (**A**) and linear (**B**) ***zot***-positive contigs identified in the current study. Open reading frames (ORFs) are represented by arrows

Four of nine complete zot genes identified in the current study were carried on circular contigs, which likely represent plasmid-like replicative forms of complete FPP genomes. According to the designations proposed by Narancic et al. [17], the FPPs predicted in our isolates were designated as AfM2, AfM11, AfM15, and AfM18. Two isolates, M9 and M12, harbored two copies of the *zot* gene located on linear contigs, where the surrounding genetic context could not be determined. In contrast, isolate M18 carried an additional *zot* gene embedded within a prophage-like sequence integrated into the chromosome. Circular and linear maps of the *zot*-positive contigs identified in our isolates are depicted in Fig. 1. The BLASTn analysis of the predicted FPP genomes against the nr/nt NCBI database showed identical and closely similar (100% coverage and > 99% identity) FPPs in other *A. baumannii* isolates that belong mostly to the same STs (except AfM15), as shown in Supplementary Table 2.

The chromosome-integrated FPP genome-like sequence identified in M18 showed the highest similarity to *zot*-harboring prophages integrated into the chromosomes of *A. baumannii* of the same ST including ACN21 (GenBank accession: CP038644.1) and Cl300 (GenBank accession: CP082952.1). All were inserted in the upstream region of the exonuclease ABC subunit UvrA-coding gene. While a gene coding type I fimbrial protein was found downstream of the prophages in ACN21 and Cl300, the full sequence and the complete environment of the prophage could not be determined.

### Phylogeny of the FPPs predicted in our isolates

**Fig. 2:**
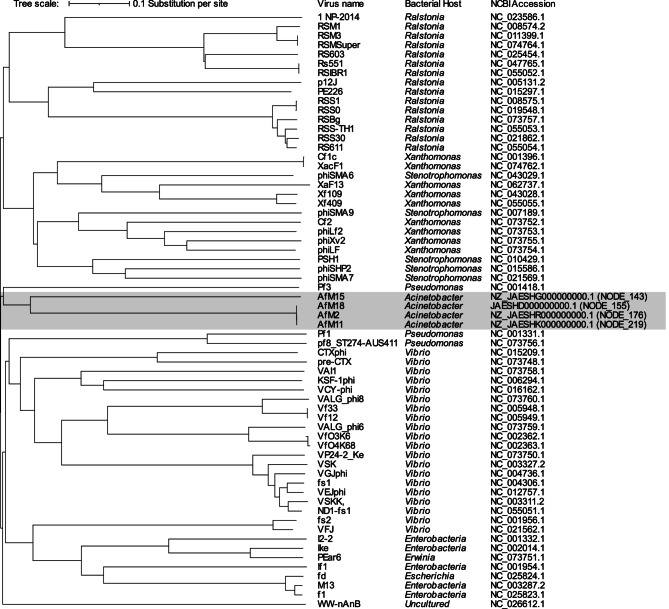
Proteome tree generated from 60 RefSeq bacteriophage genomes that belong to the family *Inoviridae* and the genomes of 4 FPPs predicted in *A. baumannii* included in the current study

A total of 382 *Inoviridae* genomic sequences were available in the NCBI genomic database at the time of analysis (accessed on April 19, 2025), including 75 RefSeq genomes. For the purpose of phylogenetic analysis, only the RefSeq sequences were considered to ensure high-quality, curated data. To avoid redundancy, in cases where multiple genomic entries existed for the same virus, only one representative sequence per virus was included in the analysis. Accordingly, 60 non-redundant RefSeq genomic sequences were selected. These were analyzed alongside the four complete circular FPP genomes identified in the current study. The resulting proteome tree indicated that the closest FP to those predicted in our *A. baumannii* isolates is the *Pseudomonas* bacteriophage Pf3, as shown in Fig. 2.

Based on the grouping scheme proposed by Narancic et al. (2024) [17] for *A. baumannii* FPs, phylogenetic analysis of the predicted amino acid sequences of the Zot proteins revealed that a total of three FPPs belonged to Group A (M02, M11, and M18); four FPPs to Group C (M06, M09, M12, and M15); two FPPs to Group E (M09 and M12); one FPP to Group F (M03); and one FPP to Group G (M18). These classifications are supported by the phylogenetic tree shown in Supplementary Fig. 2.

### ST/GC distribution of FPPs in *A. baumannii* deposited in the NCBI genomic database

Searching the NCBI database for the *zot*-positive *A. baumannii* isolates, a total of 126 complete genomes were found. The strain names, STs, and the GenBank accession numbers of their genomic sequences are listed in Supplementary Table 3. These were found to belong to different sequence types (42 Pasteur STs and 66 Oxford STs). A minimum spanning tree was created using the Pasteur STs of the *zot*-positive *A. baumannii* retrieved from the NCBI and the isolates collected in the current study (Supplementary Fig. 3). Based on the clonal complexes assigned for the STs to which the *zot*-positive strains belong, we found that FPPs are disseminated in at least six GCs, namely GC1, GC2, GC4, GC7, GC9, and GC11 [41–45]. Most commonly, they belonged to GC1 (26.0% of the isolates).

### Biofilm phenotypes among *zot*-positive and *zot*-negative isolates

Based on their BFIs, *A. baumannii* isolates in the present study were classified into strong (33.3%), moderate (27.7%), weak (33.3%), and non-biofilm forming (5.0%). While none of the *zot*-negative isolates could form strong biofilms, six (66.6%) out of the nine *zot*-positive isolates formed strong biofilms. The biofilm phenotypes of all isolates are shown in Table 2.

**Table 2:** Biofilm formation phenotypes of all isolates

Isolate number	ST^Oxf^	ST^Pas^	GC	zot gene(s)	BFI	Biofilm Phenotype
M1	1050/2058	2	2	Negative	0.1	Negative
M2	1089	85	9	Positive	6.8	Strong
M3	22,461	113	7	Positive	4.1	Strong
M4	1816/195	2	2	Negative	1.0	Moderate
M5	1816/195	2	2	Negative	1.5	Moderate
M6	1604/231	ND	1	Positive	2.9	Strong
M9	1604/231	ND	1	Positive	0.4	Weak
M10	14,181	ND^a^	-	Negative	0.8	Weak
M11	1089	85	9	Positive	1.9	Strong
M12	1604/231	19	1	Positive	1.2	Moderate
M13	1816/195	2	2	Negative	1.7	Moderate
M14	23,291	113	7	Positive	0.4	Weak
M15	1604/231	19	1	Positive	1.9	Strong
M16	1050/2058	2	2	Negative	0.8	Weak
M17	1050/2058	2	2	Negative	1.0	Moderate
M18	1078	85	9	Positive	1.8	Strong
M19	1418	164	-	Negative	0.7	Weak
M20	1701	570	2	Negative	0.9	Weak

**Fig. 3:**
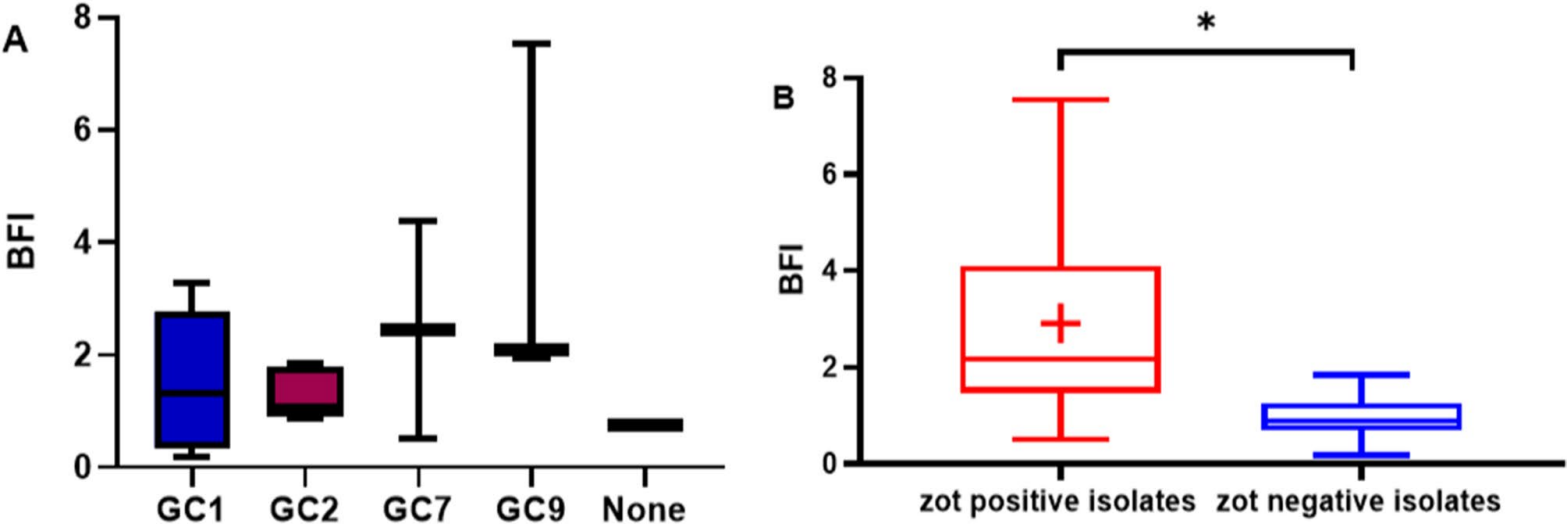
Boxplots showing the distribution of the BFIs of the ***A. baumannii*** isolates that belong to different GCs (**A**) and ***zot***-positive versus ***zot***-negative isolates (**B**). The mean and median BFI values are marked by the cross and line within the box, respectively. A statistically significant difference (*P* < 0.05) is denoted by an Asterisk (*)

Comparing the BFIs of the isolates across the two groups showed that the BFIs of the isolates carrying FPPs were significantly higher than the members of the other group (unpaired t-test, P-value = 0.0156). In the meantime, we found no significant difference between the BFIs of the isolates that belong to different GCs (One way ANOVA, P-value = 0.2252), as shown in Fig. 3.

### The potential enterotoxicity of the isolates carrying FPPs

#### Zot protein features

Using the predicted amino acid sequences of Zot proteins as inputs for the BTXpred server confirmed that all Zot proteins encoded by our isolates were potential exotoxins. The tool failed to define the exact function of any of the detected toxins. All proteins could only be identified by the support vector machine (SVM) method that is based on amino acid composition.

Multiple sequence alignment of the predicted amino acid sequences showed that the highest similarity between the Zot proteins encoded by our isolates and those of *V. cholera* N16961, *Vibrio parahaemolyticus* PMC53.7, *C. concisus* 13,826, *Neisseria meningitidis* MC58, and *P. aeruginosa* PAK was 15.0%, 18.9%, 22.7%, 26.3%, and 15.9%, respectively. Meanwhile, the similarity between the Zot proteins of our isolates ranged from 15.9 to 100%. The similarity matrix of all Zot proteins is shown in Supplementary Fig. 4.

Multiple sequence alignment of the Zot proteins encoded by *A. baumannii* isolates studied here and Zot proteins encoded by other bacterial pathogens showed that the glycine (G) residue of the Walker A motif of *A. baumannii*, *C. concisus*, and *N. meningitidis* was replaced by tyrosine (Y) in *V. cholerae*, *V. parahemolyticus*, and *P. aeruginosa*. The Zot receptor binding motif (GXXXVQXG) and the FCIGRL sequence, which is located in the C-terminal domain of Zot proteins, were not detected in any of the analyzed species except *V. cholera* (Supplementary Fig. 5).

#### Cytotoxicity of the CFSs of the *zot*-positive isolates to Caco-2 cells

**Fig. 4:**
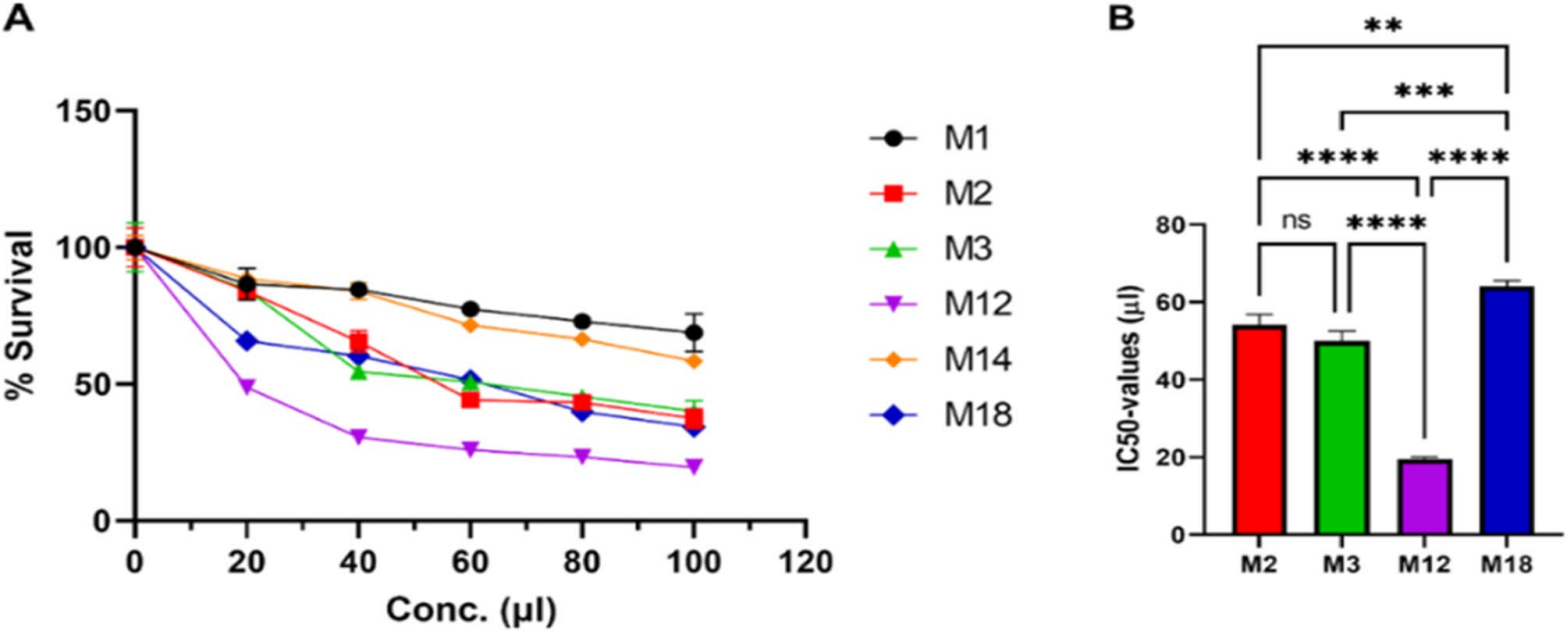
The effect of different concentrations of bacterial isolates and their negative and positive controls on the viability of Caco-2 cancer cell line by MTT assay after incubation for 48 h (**A**) and their IC50 values (**B**). The data is presented as the Means ± SD of three technical replicates for each CFS concentration

The effect of the CFSs of the *zot*-positive *A. baumannii* isolates on the viability of Caco-2 cells compared to the *zot*-negative strain, M1, was investigated by the MTT assay. The cells treated with the CFSs of the isolates M2, M3, M12, and M18 showed a significantly higher cytotoxic effect compared to the negative control M1 and M14 that carried an incomplete *zot* sequence (Fig. 4A). Moreover, M12 showed a significantly strong cytotoxic effect on the cells with a mean IC50 value of 19.44 ± 0.58 compared to M2, M3, and M18 with IC50 values equal 54.19 ± 2.66, 49.99 ± 2.55, and 64.09 ± 1.455, respectively (Fig. 4B).

As described by the ATCC, Caco-2 cells are cuboidal with a very definitive border; each cell separated by a thin space. Exposure of Caco-2 cells to 100 µL of the CFSs of M2, M3, M12, and M18 affected cells’ morphology. Many cells were detached from the surfaces of the flasks, lost their epithelial morphology, and shrank, which is typical for cells undergoing apoptosis. On the other hand, M1 and M14-treated cells maintained their epithelial morphology, showed defined borders, and remained attached to the culture flasks, as shown in Supplementary Fig. 6.

### Histopathological examination

**Fig. 5:**
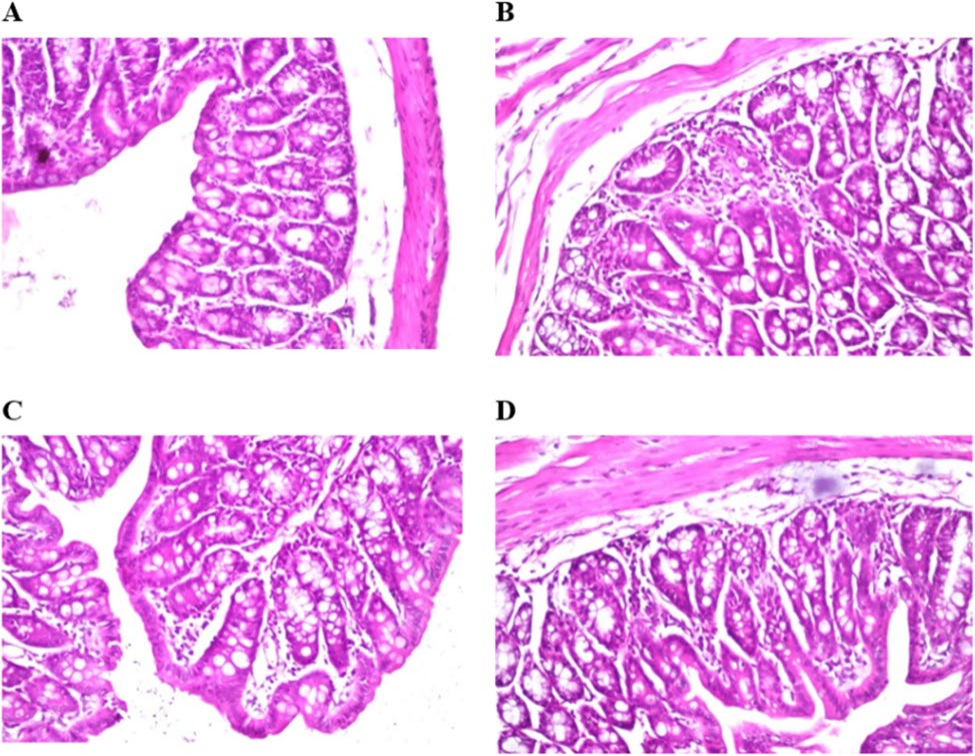
Histopathological examination of the large intestine of adult BALB/c mice 24 h after intraperitoneal injection of saline (**A**) and CFS of M12 (**B**, **C**, and **D**)

After the intraperitoneal injection of the CFS of M12 into the test group, severe diarrhea was observed within 6 h, while the control group showed no symptoms. To assess whether the toxin induces inflammatory responses in the large intestine, intestinal tissue was examined 24 h post-injection. The control group showed no histopathological alterations, maintaining the normal histological structure of the mucosa, including the lining epithelium and underlying lamina propria (Fig. 5A). In contrast, the test group exhibited significant histological changes, with the lamina propria showing focal inflammatory cell infiltration and edema beneath the lining mucosal epithelium (Fig. 5B). Additionally, inflammatory cell infiltration was noted in the deep lamina propria between the glands (Fig. 5C), and a few inflammatory cells with edema were detected in the submucosa (Fig. 5D).

## Discussion

While extensive research has focused on understanding the genetic mechanisms underlying antibiotic resistance in *A. baumannii*, the presence and impact of prophages, specifically FPPs, in this pathogen have remained largely unexplored. The current study aimed to explore the existence of FPPs in *A. baumannii* genomes and their potential impact on virulence. To achieve our objectives, we employed a combined approach of bioinformatics analysis and phenotypic characterization of clinical isolates of *A. baumannii* collected from patients in Egypt in 2020 in a previous study [18, 46]. Most of the isolates belonged to GCs that are widely disseminated in Egypt [47, 48] and worldwide [49–51].

Being conserved in all FPs, *zot* genes were used as markers for our screening, as described before [17, 52]. The gene was found in 9/18 (50%) of our isolates. Complete *zot* gene sequences were identified in the genomes of 6 isolates (M2, M9, M11, M12, M15, and M18), while only partial sequences were identified in M3 and M6. The *zot* gene carried by M14 was interrupted by an unknown genetic element. Based on the topology identified by Bandage, the complete *zot* genes were found to be embedded in circular (M2, M11, M15, and M18) as well as linear (M9, M12, and M18) contigs. The circular contigs were predicted as extrachromosomal plasmid-like forms of complete genomes of FPPs that were designated as AfM2, AfM11, AfM15, and AfM18.

The *zot*-positive contigs of M9 and M12 were predicted as tandem repeats of FPPs carrying two copies of the FPP signature genes, except the major coat protein, which was present in only one copy. Tandem repeats of FPP in bacterial chromosomes have been previously described in *V. cholerae* CTXφ by Davis and Waldor (2000) study [53]. The authors suggested that the presence of tandem elements is necessary for the production of virions, and that *V. cholerae* isolates with a solitary prophage seldom produce CTXφ virions. Finally, M18 was predicted to carry two different FPPs, the first one exists in a plasmid-like format (AfM18) while the other is integrated within the chromosome.

In the study by Narancic et al. (2024) [17], in silico analysis predicted the size of the FPP Af1 in *A. baumannii* ATCC 19,606 to be 7201 nucleotides. However, gel electrophoresis of the Af1 genome performed by the authors suggested a size range between 6,557 and 9,416. This variation is likely due to the circular single-stranded nature of the Af1 genome, which results in a different migration pattern compared to the linear double-stranded DNA ladder used in electrophoresis. The circular forms of the FPPs identified in this study varied in size from 5,816 to 6,697 bases. Notably, the PHASTER online tool failed to identify the FPPs in the genomes of the *zot*-positive contigs. False-negative results for the identification of FPPs using PHASTER were also reported before [54]. This was assumed to result from their short genome and the reliance of the software on information about previously identified viruses [54].

While Narancic et al. (2024) [17] have investigated the prevalence of the *zot* gene (representing FPPs) in the genomes of *A. baumannii* deposited in the NCBI genomic database; they did not explore the most similar inoviruses to those of *A. baumannii* or the ST/GC distribution of FPP in *A. baumannii*. In the present study, a proteome tree was generated to investigate the phylogeny of the FPPs predicted in this research, in relation to all RefSeq genomic sequences belonging to the *Inoviridae* family. Interestingly, our predicted FPPs clustered with *P. aeruginosa* Pf3. One possible explanation is the relatively close taxonomic relationship between *A. baumannii* and *P. aeruginosa*, as both belong to the class Gammaproteobacteria within the order Pseudomonadales. This phylogenetic proximity may promote the exchange or conservation of prophage genetic elements. Additionally, their ecological overlap and frequent co-occurrence in both clinical and environmental settings may facilitate cross-genus horizontal transfer of FPs, which could account for the observed clustering with *P. aeruginosa* Pf3.

Narancic et al. (2024) [17] proposed a classification system for FPs based on the predicted amino acid sequences of Zot proteins, identifying at least ten distinct groups, designated A through J. By comparing the predicted amino acid sequences identified in the current study with representative Zot proteins from each group, the FPPs in our isolates were successfully assigned to specific groups. The majority of FPPs clustered within Group C, while others were classified A, E, F, and G.

Exploring the ST/GC of the FPP-harboring isolates revealed their dissemination in 42 Pasteur STs spanning at least six GCs, most commonly GC1. *A. baumannii* GCs are generally associated with higher antimicrobial resistance and worse disease outcomes. Hence, one of our aims in the current study was to re-evaluate the impact of FPPs on antimicrobial resistance and biofilm formation by making comparisons between FPP-positive and FPP-negative isolates. Based on our previous study [18], the 18 isolates examined here are multidrug-resistant (MDR) or extensively drug-resistant (XDR). While the infection studies conducted by Narancic et al. (2024) [17] on *zot*-negative *A. baumannii* strains demonstrated that FP infection was associated with increased susceptibility to specific groups of antimicrobial agents, their work focused solely on FPs belonging to groups A and C. In contrast, the FPPs identified in the current study also include members from other groups. Therefore, we recommend that future studies investigate the impact of different groups of FPs on the antimicrobial susceptibility profiles across various classes of antimicrobial agents, to determine whether such effects are group-specific.

The results of the current study also showed that the majority of the *zot*-positive isolates demonstrated strong biofilm formation, while none of the *zot*-negative isolates were capable of forming strong biofilms. Furthermore, a comparison of the BFIs between the two groups indicated that the isolates carrying FPPs had significantly higher BFIs than the others. No significant differences in the BFIs were found among the isolates belonging to different GCs. Earlier studies have shown that FPs promote biofilm formation by *P. aeruginosa* and that the production of FP in *P. aeruginosa* biofilms is associated with a liquid crystalline biofilm matrix that also promotes persistence through providing desiccation and antibiotic tolerance [11]. Compared to planktonic bacteria, genes encoding FPs were found to be the most upregulated in *P. aeruginosa* biofilms [55]. It is worth mentioning that the infection studies conducted by Narancic et al. (2024) [17] demonstrated that FP infection in *zot*-negative *A. baumannii* strains was associated with reduced biofilm formation. As mentioned earlier, their investigation involved only two FP groups (Group A and Group C). Thus, broader infection studies including FPs from additional groups are recommended to determine whether the observed effects on biofilm formation are group-specific. Moreover, in their biofilm assay, Narancic et al. (2024) [17] did not calculate the biofilm index (the ratio of OD_biofilm_ to OD_growth_), which corrects for differences in bacterial growth. Since their study also reported a reduced growth rate in FP-infected strains, the observed decrease in biofilm biomass may, at least in part, reflect reduced bacterial growth rather than a specific impairment of biofilm formation. However, it is also important to note that the findings related to biofilm formation in the current study are limited by the relatively small number of isolates examined. Future association studies incorporating a larger sample number of *A. baumannii* strains and a broader representation of isolates from different GCs are necessary to validate these findings. Additionally, infection studies of *zot*-negative *A. baumannii* strains that include a wider range of FP groups and employ biofilm index calculations are needed to more accurately assess the impact of FP infection on biofilm formation.

To the best of our knowledge, this study is the first to explore the potential association between FPPs and the enterotoxicity of *A. baumannii.* While enough research linking *A. baumannii* to foodborne infections is lacking, evidence of the ability of *Acinetobacter* species to colonize the digestive tract abundantly exists [56–58]. Evidence on the enterotoxigenic potential of *A. baumannii* was provided by Polanco and Manzi (2008) [59] who isolated the pathogen from the feces of young children with acute diarrhea. The authors also demonstrated the cytotoxicity of the isolates to cell cultures. In a later study by Thom et al. (2010) [60], the majority of patients with bloodstream infections (BSIs) were found to be previously colonized in the gastrointestinal tract (GIT) with genetically similar isolates. This was evidenced by pulsed-field gel electrophoresis. The authors, hence, proposed that BSIs caused by *A. baumannii* may be preceded by gut colonization. Recently, Ye et al. (2019) [61] described a case of community-acquired enterogenic sepsis due to *A. baumannii* in a 73-year-old patient. The patient was admitted to the emergency room with fever and gastrointestinal symptoms. The authors speculated that the organism might have accessed the bloodstream through the GIT causing severe septic shock. Additionally, *A. baumannii* has previously been isolated from various food products [62–64], emphasizing the importance of studying the enterotoxigenic potential of this species.

We employed both in silico and experimental approaches to investigate the enterotoxic potential of the Zot proteins predicted from the genomes of *A. baumannii*. With no assigned function, all Zot proteins encoded by our isolates were predicted as exotoxins by the BTXpred server. Using the same tool, Zot proteins of other bacterial pathogens including *C. concisus* 13,826 and some isolates of *Vibrio parahemolyticus* were previously identified as exotoxins, while *V. parahemolyticus* PMC53.7-encoded Zot was predicted to be an endotoxin. Only Zot of *N. meningitidis* MC58 was not predicted to be a bacterial toxin [37]. While the same study reported that *V. cholerae* N16961 Zot is an endotoxin, repeating the analysis of the same protein in the current study predicted it to be an exotoxin.

Of all Zot proteins produced by other tested bacterial pathogens, Zot proteins encoded by our isolates showed the highest similarity to that produced by *N. meningitidis* MC58 (26.3%) and *C. concisus* (22.7%). On the other hand, only 15% similarity was shown to Zot proteins from *V. cholerae*. It has been suggested before that the structure, rather than the sequence, is responsible for the biological effects of Zot on the epithelial barrier [65]. This was further confirmed by the 3D structure modeling of *V. parahemolyticus* PMC53.7 Zot performed by Perez-Reytor et al. (2020) [37]. Although *C. concisus* Zot and *V. cholerae* Zot have only 16% amino acid identity, the toxic effects of *C. concisus* Zot on the intestinal epithelial cells have been previously confirmed [66, 67]. An amino acid identity percent of 21.4 was found between Zot of *V. cholerae* and the cytotoxic strain *V. parahemolyticus* PMC53.7. Interestingly, Perez-Reytor et al. (2020) [37] reported that infection by PMC53.7 disrupted the actin cytoskeleton in Caco-2 cells.

The motif analysis of Zot proteins from various pathogenic bacteria, previously conducted by Perez-Reytor et al. (2020) [37], was extended to include the Zot proteins predicted in our isolates. Based on our analysis and as reported before [37], the Zot proteins of *A. baumannii*, *N. meningitidis*, and *C. concisus* retained the Walker A motif structure GxxxxGK[S/T], while the Zot proteins of *V. cholerae*, *V. parahaemolyticus*, and *P. aeruginosa* were found to exhibit the motif structure GxxxxYK[S/T]. Walker A and B motifs are characteristic of proteins in the P-loop NTPase superfamily and are involved in binding ATP or GTP [39]. It has been reported that the P-loop in NTPases can influence focal adhesion and actin fibers in cells [68]. Further analysis showed that the FCIGRL sequence, which has previously been recognized as the active fragment of Zot in *V. cholerae* [9, 69] was uniquely identified in the Zot protein of this species. In their study, Perez-Reytor et al. (2020) [37] have concluded that the presence of this peptide sequence is not strictly necessary for the activity of all Zot proteins.

The significant Caco-2 cell cytotoxicity caused by the CFSs of *zot*-positive *A. baumannii* isolates was observed in our study. The detachment of cells caused by the *zot*-positive bacterial isolates was in accordance with Perez-Reytor et al. (2020) [37] who observed that Chilean *V. parahaemolyticus* PMC53.7-Zot strain impaired the attachment of Caco-2 cells to the plate surface due to loss of focal adhesions, though they couldn’t proof the induction of apparent cytotoxicity as they previously suspected. This could be attributed to the activation of proteinase-activated receptor 2 (PAR2) by Zot since it targets intestinal cellular junctions, inducing its disassembly and cytoskeleton remodeling [67]. Interestingly, another study suggested that *C. concisus* Zot has enteric pathogenic potential by causing prolonged damage to the intestinal epithelial barrier by inducing epithelial apoptosis as demonstrated in Caco-2 cells. It also induced intestinal epithelial and macrophage production of proinflammatory cytokines, in particular TNF-α, and enhanced the responses of macrophages in HT-29 cells [66]. Our in vivo analysis of Zot enterotoxicity showed the occurrence of severe diarrhea similar to that caused by *V. cholerae* Zot [9], and the presence of inflammatory cells with diffuse edema in the histopathological analysis was in accordance with the induced upregulation of TLR3, pro-inflammatory cytokines IL6, IL8 and chemokine CXCL16, as well as the inflammatory caspase CASP7 observed previously [67]. Similar histopathological alterations in the intestinal mucosa and submucosa were reported by Charla et al. (2022) [70], which was induced by CFS of *V. cholerae* isolated from clinical samples during cholera outbreaks in Karnataka, India.

While our in vitro experiments demonstrated that the culture supernatants of *zot*-positive *A. baumannii* isolates exhibited greater cytotoxic effects compared to a *zot*-negative control strain, and the enterotoxicity of one *zot*-positive isolate was confirmed by an in vivo study, we acknowledge that these findings do not definitively establish that the Zot protein alone is responsible for the observed phenotypes. Therefore, we recommend that future studies utilize genetic approaches, such as *zot* gene knockout or complementation assays, to directly assess and validate the specific contribution of the Zot protein to these effects.

## Conclusion

The current study highlights the presence of FPPs in clinical isolates of *A. baumannii* from Egypt and explores their potential implications for the virulence of this notorious pathogen. Our findings indicate that FPPs are disseminated among the GC isolates in our collection and within genomes retrieved from the NCBI database. We observed a significant association between FPPs and enhanced biofilm formation in *A. baumannii*. Additionally, isolates carrying FPPs exhibited increased cytotoxicity to Caco-2 cells. Intraperitoneal injection of the CFS from an FPP-positive strain into BALB/c mice resulted in severe diarrhea, accompanied by significant histological changes. Our findings contribute to the growing body of knowledge surrounding bacteriophage-host interactions. It also advances our understanding of the biology of *A. baumannii* and provides novel perspectives on the role of FPs in shaping their pathogenicity. Future studies are recommended to provide genetic evidence to support our findings. Additionally, a larger sample size of *A. baumannii* strains representing different GCs should be included in future research to validate these results. Furthermore, it is essential to investigate potential strategies to target FPs, aiming to reduce virulence and improve therapeutic outcomes.

## Supplementary Information

Supplementary Material 1.

## Data Availability

The nucleotide sequences of the FPP genomes that support the findings of this study have been deposited in the GenBank database under the BioProject number PRJNA690827. The contigs representing the zot-positive contigs are listed in Supplementary information file.

## Abbreviations

FPPs: Filamentous Prophages
GCs: Global Clones
FPs: Filamentous Phages
ST/GC: Sequence Type/Global Clone
MDR: Multidrug-Resistant
XDR: Extensively Drug-Resistant
BFI: Biofilm Formation Index
BPs: Base Pairs
NCBI: National Center for Biotechnology Information
CFS: Cell-Free Supernatant
Caco-2: Human Colorectal Adenocarcinoma Cells
IL6: Interleukin 6
IL8: Interleukin 8
CXCL16: C-X-C Motif Chemokine Ligand 16
CASP7: Caspase 7
TLR3: Toll-Like Receptor 3
