# Supplementary material for: Filamentous prophages in the genomes of Acinetobacter baumannii from egypt: impact on biofilm formation and the potential to induce enterotoxicity
Source: BMC Microbiol. 2025 Jul 23;25:449. doi: 10.1186/s12866-025-04177-z (PMC12285184; doi:10.1186/s12866-025-04177-z)
Supplement: Supplementary file 1 — Supplementary Material 1. [file 12866_2025_4177_MOESM1_ESM.docx]

Supplementary Material

**Supplementary Table 1: GenBank accession numbers of the *zot*-positive isolates collected in the current study**

| **Isolate No.** | **WGS Accession** | **Zot-positive contig names** |
| --- | --- | --- |
| M02 | NZ_JAESHR000000000.1 | NODE_176_length_6243_cov_9.777959 |
| M03 | NZ_JAESHQ000000000.1 | NODE_585_length_977_cov_2.705882 |
| M06 | JAESHN000000000.1 | NODE_692_length_1676_cov_2.537121 |
| M09 | JAESHM000000000.1 | NODE_15_length_13511_cov_10.151076 |
| M11 | NZ_JAESHK000000000.1 | NODE_219_length_5943_cov_18.084078 |
| M12 | NZ_JAESHJ000000000.1 | NODE_103_length_13396_cov_16.216444 |
| M14 | NZ_JAESHH000000000.1 | NODE_622_length_1155_cov_2.766537 |
| M15 | NZ_JAESHG000000000.1 | NODE_143_length_6824_cov_18.375243 |
| M18 | JAESHD000000000.1 | NODE_30_length_29020_cov_25.256637 |
|  |  | NODE_155_length_5943_cov_25.478851 |

**Supplementary Table 2: Top hits of the BLASTn analysis of the FPPs identified in our study against the NCBI nr/nt database**

| FP | Strain | ST^Oxf/Pas^ | coverage | identity | Accession |
| --- | --- | --- | --- | --- | --- |
| AfM2 | *Acinetobacter baumannii* strain AB177-VUB | 1089/85 | 100% | 100.00% | CP091361.1 |
|  | *Acinetobacter baumannii* strain Cl300 | 1089/85 | 100% | 99.98% | CP082952.1 |
|  | *Acinetobacter baumannii* strain AB186-VUB | 1752/85 | 100% | 99.98% | CP091356.1 |
|  | *Acinetobacter baumannii* strain ACN21 | 1089/85 | 100% | 99.90% | CP038644.1 |
|  | *Acinetobacter baumannii* strain A388 | 439/1 | 90% | 96.48% | CP024418.1 |
|  | *Acinetobacter baumannii* strain OC064 | 439/1 | 90% | 96.48% | CP087317.1 |
| AfM11 | *Acinetobacter baumannii* strain Cl300 | 1089/85 | 100% | 99.98 | [CP082952.1](https://www.ncbi.nlm.nih.gov/nucleotide/CP082952.1?report=genbank&log$=nucltop&blast_rank=1&RID=212TUT29013) |
|  | *Acinetobacter baumannii* strain AB186-VUB | 1752/85 | 100% | 99.98 | [CP091356.1](https://www.ncbi.nlm.nih.gov/nucleotide/CP091356.1?report=genbank&log$=nucltop&blast_rank=2&RID=212TUT29013) |
|  | *Acinetobacter baumannii* strain AB177-VUB | 1089/85 | 100% | 99.98 | [CP091361.1](https://www.ncbi.nlm.nih.gov/nucleotide/CP091361.1?report=genbank&log$=nucltop&blast_rank=3&RID=212TUT29013) |
|  | *Acinetobacter baumannii* strain ACN21 | 1089/85 | 100% | 99.95 | [CP038644.1](https://www.ncbi.nlm.nih.gov/nucleotide/CP038644.1?report=genbank&log$=nucltop&blast_rank=4&RID=212TUT29013) |
|  | *Acinetobacter baumannii* strain A388 | 439/1 | 94% | 97.34 | [CP024418.1](https://www.ncbi.nlm.nih.gov/nucleotide/CP024418.1?report=genbank&log$=nucltop&blast_rank=5&RID=212TUT29013) |
|  | *Acinetobacter baumannii* strain OC064 | 439/1 | 94% | 97.34 | [CP087317.1](https://www.ncbi.nlm.nih.gov/nucleotide/CP087317.1?report=genbank&log$=nucltop&blast_rank=6&RID=212TUT29013) |
| AfM18 | *Acinetobacter baumannii* strain Cl300 | 1089/85 | 100% | 99.97 | [CP082952.1](https://www.ncbi.nlm.nih.gov/nucleotide/CP082952.1?report=genbank&log$=nucltop&blast_rank=1&RID=21375M5E013) |
|  | *Acinetobacter baumannii* strain AB186-VUB | 1752/85 | 100% | 99.97 | [CP091356.1](https://www.ncbi.nlm.nih.gov/nucleotide/CP091356.1?report=genbank&log$=nucltop&blast_rank=2&RID=21375M5E013) |
|  | *Acinetobacter baumannii* strain AB177-VUB | 1089/85 | 100% | 99.97 | [CP091361.1](https://www.ncbi.nlm.nih.gov/nucleotide/CP091361.1?report=genbank&log$=nucltop&blast_rank=3&RID=21375M5E013) |
|  | *Acinetobacter baumannii* strain ACN21 | 1089/85 | 100% | 99.93 | [CP038644.1](https://www.ncbi.nlm.nih.gov/nucleotide/CP038644.1?report=genbank&log$=nucltop&blast_rank=4&RID=21375M5E013) |
|  | *Acinetobacter baumannii* strain A388 | 439/1 | 94% | 97.45 | [CP024418.1](https://www.ncbi.nlm.nih.gov/nucleotide/CP024418.1?report=genbank&log$=nucltop&blast_rank=5&RID=21375M5E013) |
|  | *Acinetobacter baumannii* strain OC064 | 439/1 | 94% | 97.45 | [CP087317.1](https://www.ncbi.nlm.nih.gov/nucleotide/CP087317.1?report=genbank&log$=nucltop&blast_rank=6&RID=21375M5E013) |
| AfM9 | *Acinetobacter baumannii* strain VB2486 | 1604-231/1 | 94% | 99.87 | [CP050403.1](https://www.ncbi.nlm.nih.gov/nucleotide/CP050403.1?report=genbank&log$=nucltop&blast_rank=1&RID=213PSCUG016) |
|  | *Acinetobacter baumannii* strain 11W359501 | 1604-231/1 | 94% | 98.03 | [CP041035.1](https://www.ncbi.nlm.nih.gov/nucleotide/CP041035.1?report=genbank&log$=nucltop&blast_rank=2&RID=213PSCUG016) |
|  | *Acinetobacter baumannii* strain AB6870155 | 781/1 | 94% | 98.07 | [CP114381.1](https://www.ncbi.nlm.nih.gov/nucleotide/CP114381.1?report=genbank&log$=nucltop&blast_rank=3&RID=213PSCUG016) |
|  | *Acinetobacter baumannii* strain NCTC13421 | 1840, 207/1 | 94% | 98.01 | [LS483472.1](https://www.ncbi.nlm.nih.gov/nucleotide/LS483472.1?report=genbank&log$=nucltop&blast_rank=4&RID=213PSCUG016) |
|  | *Acinetobacter baumannii* strain FDAARGOS_1036 | 947/1 | 94% | 98.01 | [CP066016.1](https://www.ncbi.nlm.nih.gov/nucleotide/CP066016.1?report=genbank&log$=nucltop&blast_rank=5&RID=213PSCUG016) |
|  | *Acinetobacter baumannii* AB0057 | 1840, 207/1 | 94% | 98.02 | [CP001182.2](https://www.ncbi.nlm.nih.gov/nucleotide/CP001182.2?report=genbank&log$=nucltop&blast_rank=6&RID=213PSCUG016) |
|  | *Acinetobacter baumannii* strain ATCC BAA1605 | 947/1 | 94% | 96.8 | [CP058625.1](https://www.ncbi.nlm.nih.gov/nucleotide/CP058625.1?report=genbank&log$=nucltop&blast_rank=7&RID=213PSCUG016) |
| AfM12 | *Acinetobacter baumannii* strain VB2486 | 1604-231/1 | 94% | 99.87 | [CP050403.1](https://www.ncbi.nlm.nih.gov/nucleotide/CP050403.1?report=genbank&log$=nucltop&blast_rank=1&RID=2154NPPM013) |
|  | *Acinetobacter baumannii* strain 11W359501 | 1604-231/1 | 94% | 98.04 | [CP041035.1](https://www.ncbi.nlm.nih.gov/nucleotide/CP041035.1?report=genbank&log$=nucltop&blast_rank=2&RID=2154NPPM013) |
|  | *Acinetobacter baumannii* strain AB6870155 | 781/1 | 94% | 98.08 | [CP114381.1](https://www.ncbi.nlm.nih.gov/nucleotide/CP114381.1?report=genbank&log$=nucltop&blast_rank=3&RID=2154NPPM013) |
|  | *Acinetobacter baumannii* strain NCTC13421 | 1840, 207/1 | 94% | 98.02 | [LS483472.1](https://www.ncbi.nlm.nih.gov/nucleotide/LS483472.1?report=genbank&log$=nucltop&blast_rank=4&RID=2154NPPM013) |
|  | *Acinetobacter baumannii* strain FDAARGOS_1036 | 947/1 | 94% | 98.02 | [CP066016.1](https://www.ncbi.nlm.nih.gov/nucleotide/CP066016.1?report=genbank&log$=nucltop&blast_rank=5&RID=2154NPPM013) |
|  | *Acinetobacter baumannii* AB0057 | 1840, 207/1 | 94% | 98.02 | [CP001182.2](https://www.ncbi.nlm.nih.gov/nucleotide/CP001182.2?report=genbank&log$=nucltop&blast_rank=6&RID=2154NPPM013) |
|  | *Acinetobacter baumannii* strain ATCC BAA1605 | 947/1 | 94% | 96.86 | [CP058625.1](https://www.ncbi.nlm.nih.gov/nucleotide/CP058625.1?report=genbank&log$=nucltop&blast_rank=7&RID=2154NPPM013) |
| AfM15 | *Acinetobacter baumannii* strain D36 | 498/81 | 100% | 100 | [CP012952.1](https://www.ncbi.nlm.nih.gov/nucleotide/CP012952.1?report=genbank&log$=nucltop&blast_rank=1&RID=214SFTBF016) |
|  | *Acinetobacter baumannii* strain VB2486 | 1604-231/1 | 100% | 99.99 | [CP050403.1](https://www.ncbi.nlm.nih.gov/nucleotide/CP050403.1?report=genbank&log$=nucltop&blast_rank=2&RID=214SFTBF016) |
|  | *Acinetobacter baumannii* strain 5457 | 1507, 2433/623 | 100% | 99.99 | [CP045541.1](https://www.ncbi.nlm.nih.gov/nucleotide/CP045541.1?report=genbank&log$=nucltop&blast_rank=3&RID=214SFTBF016) |
|  | *Acinetobacter baumannii* strain AB6870155 | 781/1 | 100% | 98.07 | [CP114381.1](https://www.ncbi.nlm.nih.gov/nucleotide/CP114381.1?report=genbank&log$=nucltop&blast_rank=4&RID=214SFTBF016) |
|  | *Acinetobacter baumannii* strain ARC6851 | 1567/1 | 100% | 98.05 | [CP106943.1](https://www.ncbi.nlm.nih.gov/nucleotide/CP106943.1?report=genbank&log$=nucltop&blast_rank=5&RID=214SFTBF016) |
|  | *Acinetobacter baumannii* strain UC22850 | 1604, 231/1 | 100% | 98.04 | [CP076821.1](https://www.ncbi.nlm.nih.gov/nucleotide/CP076821.1?report=genbank&log$=nucltop&blast_rank=6&RID=214SFTBF016) |
|  | *Acinetobacter baumannii* strain DA33382 | 1567/1 | 100% | 98.02 | [CP030106.1](https://www.ncbi.nlm.nih.gov/nucleotide/CP030106.1?report=genbank&log$=nucltop&blast_rank=7&RID=214SFTBF016) |
|  | *Acinetobacter baumannii* strain OC081 | 775/1 | 100% | 98.02 | [CP087304.1](https://www.ncbi.nlm.nih.gov/nucleotide/CP087304.1?report=genbank&log$=nucltop&blast_rank=8&RID=214SFTBF016) |
|  | *Acinetobacter baumannii* strain WCHAB005078 | 449/20 | 100% | 98.01 | [CP027246.2](https://www.ncbi.nlm.nih.gov/nucleotide/CP027246.2?report=genbank&log$=nucltop&blast_rank=9&RID=214SFTBF016) |
|  | *Acinetobacter baumannii* strain 11W359501 | 1604-231/1 | 100% | 98.01 | [CP041035.1](https://www.ncbi.nlm.nih.gov/nucleotide/CP041035.1?report=genbank&log$=nucltop&blast_rank=10&RID=214SFTBF016) |
|  | *Acinetobacter baumannii* strain NCTC13421 | 1840, 207/1 | 100% | 98.01 | [LS483472.1](https://www.ncbi.nlm.nih.gov/nucleotide/LS483472.1?report=genbank&log$=nucltop&blast_rank=11&RID=214SFTBF016) |
|  | *Acinetobacter baumannii* strain AB169-VUB | 2323, 405/1 | 100% | 98.01 | [CP091367.1](https://www.ncbi.nlm.nih.gov/nucleotide/CP091367.1?report=genbank&log$=nucltop&blast_rank=12&RID=214SFTBF016) |
|  | *Acinetobacter baumannii* strain FDAARGOS_1036 | 947/1 | 100% | 98.01 | [CP066016.1](https://www.ncbi.nlm.nih.gov/nucleotide/CP066016.1?report=genbank&log$=nucltop&blast_rank=13&RID=214SFTBF016) |
|  | *Acinetobacter baumannii* strain AB322 | 1604, 231/1 | 100% | 98.01 | [CP119232.1](https://www.ncbi.nlm.nih.gov/nucleotide/CP119232.1?report=genbank&log$=nucltop&blast_rank=14&RID=214SFTBF016) |
|  | *Acinetobacter baumannii* AB0057 | 1840, 207/1 | 100% | 98.02 | [CP001182.2](https://www.ncbi.nlm.nih.gov/nucleotide/CP001182.2?report=genbank&log$=nucltop&blast_rank=15&RID=214SFTBF016) |
|  | *Acinetobacter baumannii* strain A388 | 439/1 | 100% | 99.94 | [CP024418.1](https://www.ncbi.nlm.nih.gov/nucleotide/CP024418.1?report=genbank&log$=nucltop&blast_rank=16&RID=214SFTBF016) |
|  | *Acinetobacter baumannii* strain ATCC BAA1605 | 947/1 | 100% | 97.69 | [CP058625.1](https://www.ncbi.nlm.nih.gov/nucleotide/CP058625.1?report=genbank&log$=nucltop&blast_rank=17&RID=214SFTBF016) |
|  | *Acinetobacter baumannii* strain AB5075 | 1677, 945/1 | 96% | 97.97 | [CP113078.1](https://www.ncbi.nlm.nih.gov/nucleotide/CP113078.1?report=genbank&log$=nucltop&blast_rank=18&RID=214SFTBF016) |
|  | *Acinetobacter baumannii* strain AB5075-T | 1677, 945/1 | 96% | 97.97 | [CP113080.1](https://www.ncbi.nlm.nih.gov/nucleotide/CP113080.1?report=genbank&log$=nucltop&blast_rank=19&RID=214SFTBF016) |
|  | *Acinetobacter baumannii* strain AB5075-UW | 1677, 945/1 | 96% | 97.97 | [CP008706.1](https://www.ncbi.nlm.nih.gov/nucleotide/CP008706.1?report=genbank&log$=nucltop&blast_rank=20&RID=214SFTBF016) |
|  | *Acinetobacter baumannii* strain AB5075-VUB | 1677, 945/1 | 96% | 97.97 | [CP070362.2](https://www.ncbi.nlm.nih.gov/nucleotide/CP070362.2?report=genbank&log$=nucltop&blast_rank=21&RID=214SFTBF016) |
|  | *Acinetobacter baumannii* strain AB5075-VUB-itrA::ISAba13 | 1677, 945/1 | 96% | 97.94 | [CP070358.2](https://www.ncbi.nlm.nih.gov/nucleotide/CP070358.2?report=genbank&log$=nucltop&blast_rank=22&RID=214SFTBF016) |
|  | *Acinetobacter baumannii* strain OC074 | 33 (Pas) | 90% | 95.14 | [CP087328.1](https://www.ncbi.nlm.nih.gov/nucleotide/CP087328.1?report=genbank&log$=nucltop&blast_rank=23&RID=214SFTBF016) |
|  | *Acinetobacter baumannii* strain AB43 | 705/132 | 90% | 95.14 | [CP083181.1](https://www.ncbi.nlm.nih.gov/nucleotide/CP083181.1?report=genbank&log$=nucltop&blast_rank=24&RID=214SFTBF016) |

**Supplementary Table 3: *A. baumannii* strains carrying *zot* genes in the NCBI genomic database**

| Strain name | Accession-number | ST^Oxf^ | ST^Pas^ |
| --- | --- | --- | --- |
| *Acinetobacter baumannii* strain AB0057 | CP001182.2 | 1840, 207 | 1 |
| *Acinetobacter baumannii* strain AB030 | CP009257.1 | 758 | 79 |
| *Acinetobacter baumannii* strain AbH12O-A2 | CP009534.1 | 924 | 79 |
| *Acinetobacter baumannii* strain 6200 | CP010397.1 | 1161 | 464 |
| *Acinetobacter baumannii* strain XH858 | CP014528.1 | 642 | 23 |
| *Acinetobacter baumannii* strain ab736 | CP015121.1 | 931 | 52 |
| *Acinetobacter baumannii* strain A1296 | CP018332.1 | 1469 | 138 |
| *Acinetobacter baumannii* strain USA15 | CP020595.1 | 491 | 1 |
| *Acinetobacter baumannii* strain B8342 | CP021342.1 | 2203 | 1545 |
| *Acinetobacter baumannii* strain B8300 | CP021347.1 | 2202 | 1 |
| *Acinetobacter baumannii* strain A85 | CP021782.1 | 781 | 1 |
| *Acinetobacter baumannii* strain 7804 | CP022283.1 | 490 | 25 |
| *Acinetobacter baumannii* strain A388 | CP024418.1 | 439 | 1 |
| *Acinetobacter baumannii* strain 810CP | CP026338.1 | 758 | 156 |
| *Acinetobacter baumannii* strain AR_0070 | CP027178.1 | 1627 | 32 |
| *Acinetobacter baumannii* strain AR_0052 | CP027183.1 | 1627 | 32 |
| *Acinetobacter baumannii* strain WCHAB005078 | CP027246.2 | 449 | 20 |
| *Acinetobacter baumannii* strain AR_0083 | CP027528.1 | 1604, 231 | 1 |
| *Acinetobacter baumannii* strain DA33382 | CP030106.1 | 1567 | 1 |
| *Acinetobacter baumannii* strain ATCC 17978 | CP033110.1 | 112 | 437 |
| *Acinetobacter baumannii* strain FDAARGOS_540 | CP033754.1 | 2198 | 1542 |
| *Acinetobacter baumannii* strain FDAARGOS_533 | CP033768.1 | ND | 57 |
| *Acinetobacter baumannii* strain MRSN15313 | CP033869.1 | 233 | 79 |
| *Acinetobacter baumannii* strain ACN21 | CP038644.1 | 1089 | 85 |
| *Acinetobacter baumannii* strain 11W359501 | CP041035.1 | 1604, 231 | 1 |
| *Acinetobacter baumannii* strain 5457 | CP045541.1 | 1507, 2433 | 623 |
| *Acinetobacter baumannii* strain XH1056 | CP045645.1 | ND | 256 |
| *Acinetobacter baumannii* strain DETAB-P2 | CP047973.1 | 2209 | 138 |
| *Acinetobacter baumannii* strain ABF9692 | CP048827.1 | 761 | 23 |
| *Acinetobacter baumannii* strain VB2486 | CP050403.1 | 1604, 231 | 1 |
| *Acinetobacter baumannii* strain Ab-C63 | CP051866.1 | 1552 | 107 |
| *Acinetobacter baumannii* strain Ab-D10a-a | CP051869.1 | 514 | 103 |
| *Acinetobacter baumannii* strain Ab-B004d-c | CP051875.1 | 514 | 103 |
| *Acinetobacter baumannii* strain DT0544C | CP053215.1 | 1325 | 374 |
| *Acinetobacter baumannii* strain ATCC 19606 | CP058289.1 | 931 | 52 |
| *Acinetobacter baumannii* strain ATCC BAA1605 | CP058625.1 | 947 | 1 |
| *Acinetobacter baumannii* strain AC1633 | CP059300.1 | 2089 | 126 |
| *Acinetobacter baumannii* strain 17-84 | CP059479.1 | 2142 | 63 |
| *Acinetobacter baumannii* strain XH1344 | CP061541.1 | ND | ND |
| *Acinetobacter baumannii* strain RM8376 | CP064375.1 | 931 | 52 |
| *Acinetobacter baumannii* strain ATCC 17961 | CP065432.1 | 931 | 438 |
| *Acinetobacter baumannii* strain FDAARGOS_917 1 | CP065887.1 | 931 | 307 |
| *Acinetobacter baumannii* strain FDAARGOS_1036 | CP066016.1 | 947 | 1 |
| *Acinetobacter baumannii* strain AB5075-VUB-itrA::ISAba13 | CP070358.2 | 1677, 945 | 1 |
| *Acinetobacter baumannii* strain AB5075-VUB | CP070362.2 | 1677, 945 | 1 |
| *Acinetobacter baumannii* strain KSK Sensitive | CP072305.1 | ND | 374 |
| *Acinetobacter baumannii* strain UC23022 | CP076812.1 | 1283 | 79 |
| *Acinetobacter baumannii* strain UC21460 | CP076814.1 | 225 | 318 |
| *Acinetobacter baumannii* strain UC22850 | CP076821.1 | 1604, 231 | 1 |
| *Acinetobacter baumannii* strain DETAB-E51 | CP077830.1 | 1806, 208 | 2 |
| *Acinetobacter baumannii* strain DETAB-P43 | CP077832.1 | 1837, 369 | 2 |
| *Acinetobacter baumannii* strain 17978UN | CP079931.1 | 112 | 437 |
| *Acinetobacter baumannii* strain ARLG_6344 | CP081139.1 | 1839, 281 | 2 |
| *Acinetobacter baumannii* strain Cl300 | CP082952.1 | 1089 | 85 |
| *Acinetobacter baumannii* strain AB43 | CP083181.1 | 705 | 132 |
| *Acinetobacter baumannii* strain DSM30011-VUB | CP091333.1 | 1113 | 738 |
| *Acinetobacter baumannii* strain ATCC19606-VUB | CP091334.1 | 931 | 52 |
| *Acinetobacter baumannii* strain ATCC17978-VUB | CP091335.1 | 112 | 437 |
| *Acinetobacter baumannii* strain AB231-VUB | CP091338.1 | 2056 | 215 |
| *Acinetobacter baumannii* strain AB186-VUB | CP091356.1 | 1752 | 85 |
| *Acinetobacter baumannii* strain AB177-VUB | CP091361.1 | 1089 | 85 |
| *Acinetobacter baumannii* strain AB169-VUB | CP091367.1 | 2323, 405 | 1 |
| *Acinetobacter baumannii* strain 6080 | CP096681.1 | 1806, 208 | 2 |
| *Acinetobacter baumannii* strain 280820 | CP098791.1 | 2089 | 126 |
| *Acinetobacter baumannii* strain VB280821 | CP098795.1 | 2089 | 126 |
| *Acinetobacter baumannii* strain 17978R | CP099855.1 | 112 | 437 |
| *Acinetobacter baumannii* strain 17978S | CP099856.1 | 112 | 437 |
| *Acinetobacter baumannii* strain AB105 | CP103338.1 | ND | 2266 |
| *Acinetobacter baumannii* strain ARC6851 | CP106943.1 | 1567 | 1 |
| *Acinetobacter baumannii* strain NY13623 | CP106988.1 | 1839, 281 | 2 |
| *Acinetobacter baumannii* strain 86II/2C | CP113077.1 | 1454 | 690 |
| *Acinetobacter baumannii* strain AB5075 | CP113078.1 | 1677, 945 | 1 |
| *Acinetobacter baumannii* strain AB5075-T | CP113080.1 | 1677, 945 | 1 |
| *Acinetobacter baumannii* strain AB322 | CP119232.1 | 1604, 231 | 1 |
| *Acinetobacter baumannii* strain RAB57 | CP121570.1 | 234 | 164 |
| *Acinetobacter baumannii* strain MRSN571146 | CP130627.2 | 231,1604 | 1 |
| *Acinetobacter baumannii* strain MRSN576822 | CP130628.2 | 231,1604 | 1 |
| *Acinetobacter baumannii* strain 2023CK-00127 | CP131950.1 | 281,1839 | 2 |
| *Acinetobacter baumannii* strain A207 | CP132915.1 | 2220 | 152 |
| *Acinetobacter baumannii* strain XH1044 | CP134555.1 | 1510 | 719 |
| *Acinetobacter baumannii* strain XH1041 | CP134562.1 | 642 | 23 |
| *Acinetobacter baumannii* strain XH1032 | CP134583.1 | 1508 | 108 |
| *Acinetobacter baumannii* strain ABO21-A045 | CP136178.1 | ND | 1384 |
| *Acinetobacter baumannii* strain SNUBHAB0137 | CP137055.1 | ND | 756 |
| *Acinetobacter baumannii* strain 2023CK-00001 | CP137927.1 | 281,1839 | 2 |
| *Acinetobacter baumannii* strain 2023CK-00003 | CP137930.1 | 281,1839 | 2 |
| *Acinetobacter baumannii* strain Nord12-3 | CP139831.1 | 441,1567 | 1 |
| *Acinetobacter baumannii* strain B84 | CP139836.1 | 1089 | 85 |
| *Acinetobacter baumannii* strain 2023CK-00893 | CP140420.1 | 1552 | 1142 |
| *Acinetobacter baumannii* strain 2023CK-01274 | CP140434.1 | 281,1839 | 2 |
| *Acinetobacter baumannii* strain 2023CK-01292 | CP140438.1 | 281,1839 | 2 |
| *Acinetobacter baumannii* strain 38 | CP142393.1 | 440 | 307 |
| *Acinetobacter baumannii* strain D20AB01 | CP142642.1 | 862 | 149 |
| *Acinetobacter baumannii* strain C20AB12 | CP142667.1 | 491 | 1 |
| *Acinetobacter baumannii* strain C20AB05 | CP143262.1 | 491 | 1 |
| *Acinetobacter baumannii* strain 2023CK-01512 | CP143348.1 | 281,1839 | 2 |
| *Acinetobacter baumannii* strain G20AB22 | CP146812.1 | 2929 | 1 |
| *Acinetobacter baumannii* strain AB7276 | CP149800.1 | ND | 1336 |
| *Acinetobacter baumannii* strain 2024CK-00250 | CP149831.1 | 281,1839 | 2 |
| *Acinetobacter baumannii* strain 2024CK-00130 | CP149838.1 | 2679 | 2512 |
| *Acinetobacter baumannii* strain NCSR_106 | CP154372.1 | 491 | 1 |
| *Acinetobacter baumannii* strain 2024CK-00278 | CP155455.1 | 944,1961 | 78 |
| *Acinetobacter baumannii* strain 2024CK-00329 | CP155458.1 | 281,1839 | 2 |
| *Acinetobacter baumannii* strain 2024CK-00247 | CP155476.1 | 281,1839 | 2 |
| *Acinetobacter baumannii* strain 2024CK-00246 | CP155721.1 | 944,1961 | 78 |
| *Acinetobacter baumannii* strain D13 | CP156044.1 | 231,1604 | 1 |
| *Acinetobacter baumannii* strain 2024CK-00546 | CP156933.1 | 944,1961 | 78 |
| *Acinetobacter baumannii* strain 2024CK-00462 | CP157257.1 | 281,1839 | 2 |
| *Acinetobacter baumannii* strain AB37-AUFP | CP160130.1 | 1580 | 85 |
| *Acinetobacter baumannii* strain SIMBA089 | CP162145.1 | ND | 1 |
| *Acinetobacter baumannii* strain SIMBA113 | CP162300.1 | ND | 374 |
| *Acinetobacter baumannii* strain 2024CK-00647 | CP166162.1 | 281,1839 | 2 |
| *Acinetobacter baumannii* strain AMA205 | CP169298.1 | 758 | 79 |
| *Acinetobacter baumannii* strain T14 | CP171396.1 | ND | ND |
| *Acinetobacter baumannii* strain T24 | CP171398.1 | ND | ND |
| *Acinetobacter baumannii* strain 2024CK-01407 | CP174007.1 | 1479 | 132 |
| *Acinetobacter baumannii* strain G7 | CP175642.1 | 231,1604 | 1 |
| *Acinetobacter baumannii* strain HUMC1 | CP175646.1 | 1839 | 2 |
| *Acinetobacter baumannii* strain UMB001 | CP175656.1 | 349,1838 | 2 |
| *Acinetobacter baumannii* strain A297 | CP178354.1 | 231,1604 | 1 |
| *Acinetobacter baumannii* strain PR7 | CP179694.1 | 1283 | 79 |
| *Acinetobacter baumannii* strain 2024CK-01696 | CP180034.1 | 281,1839 | 2 |
| *Acinetobacter baumannii* strain 2024CK-01880 | CP181411.1 | 281,1839 | 2 |
| *Acinetobacter baumannii* strain R2091 | LN997846.1 | 819 | 126 |
| *Acinetobacter baumannii* strain AB6870155 | NZ_CP114381.1 | 781 | 1 |
| *Acinetobacter baumannii* strain NCTC13421 1 | NZ_LS483472.1 | 207,1840 | 1 |

ND, Not defined


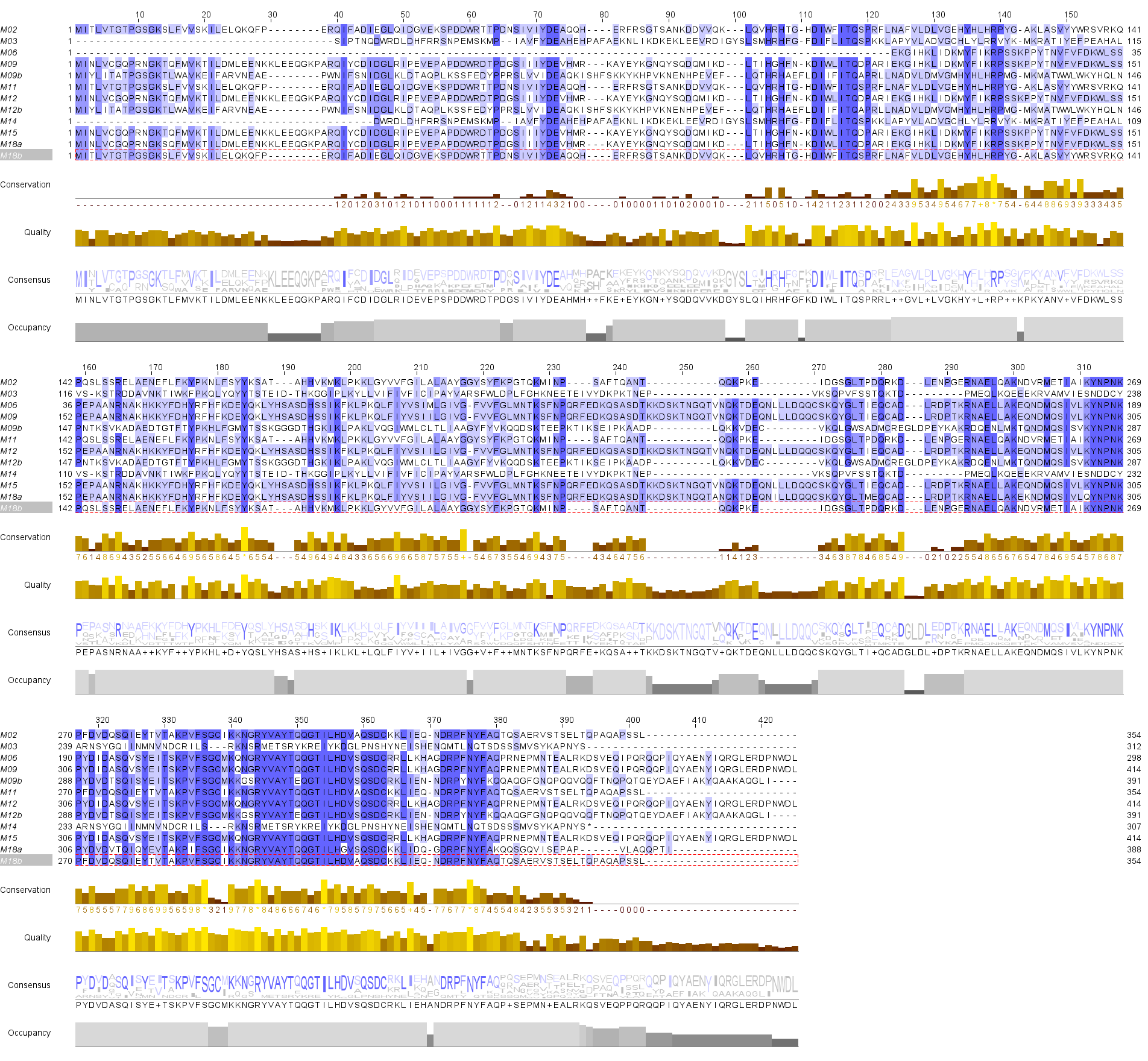


**Supplementary Figure 1: MSA of Zot proteins encoded by *A. baumannii* from the current study.** Color intensity represents the percent identity.


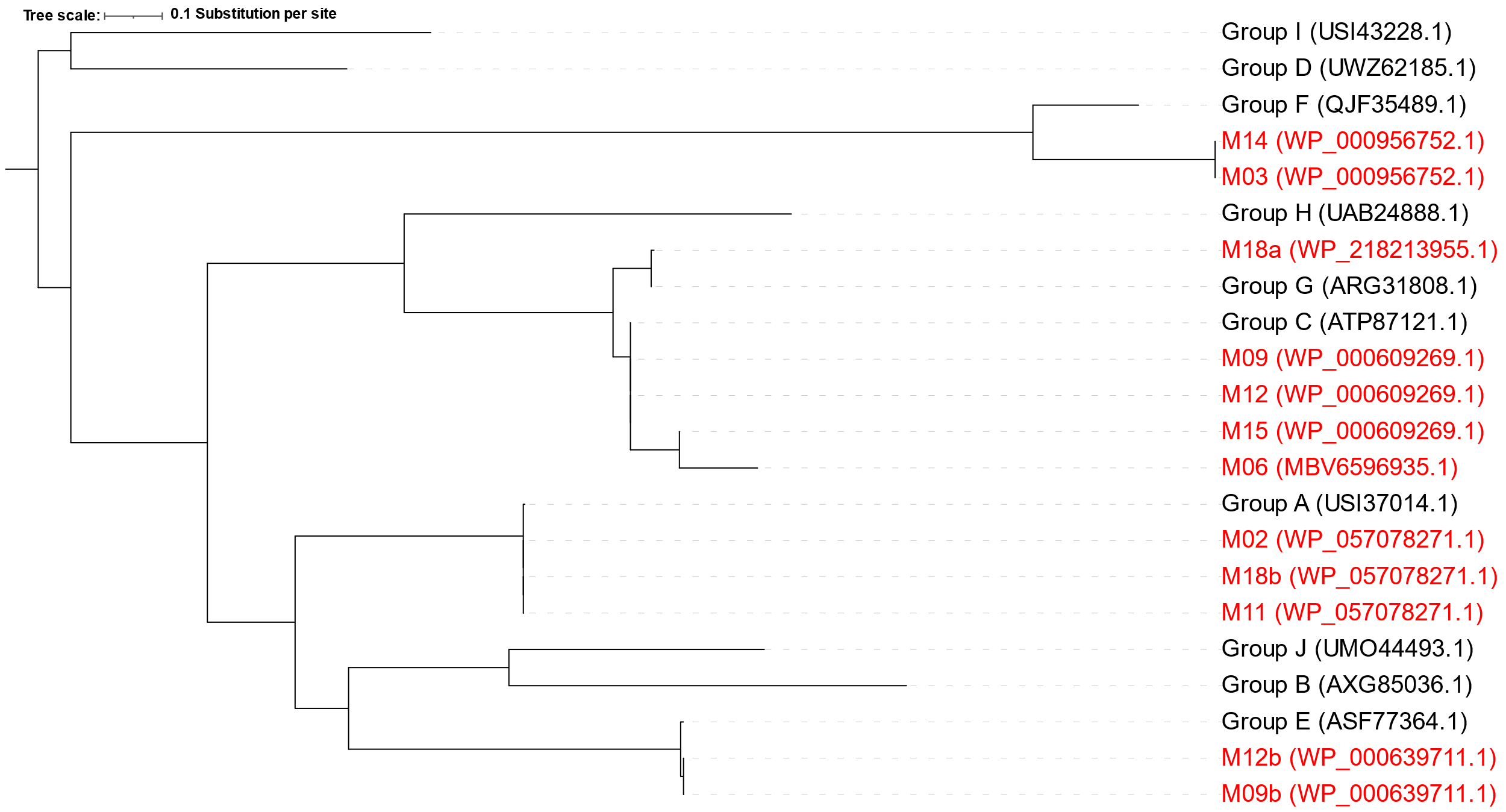


**Supplementary Figure 2: Maximum likelihood phylogenetic tree of the predicted amino acid sequences of the Zot proteins encoded by *A. baumannii* FPs identified in the current study, along with representative Zot proteins from Groups A–J as proposed by Narancic et al. (2024) [1] for classifying *A. baumannii*-specific FPs.** Protein accession numbers are indicated in brackets. Sequences identified in the current study are highlighted in red font. A lowercase letter "b" following an isolate name indicates the second FP identified within the same isolate.

**
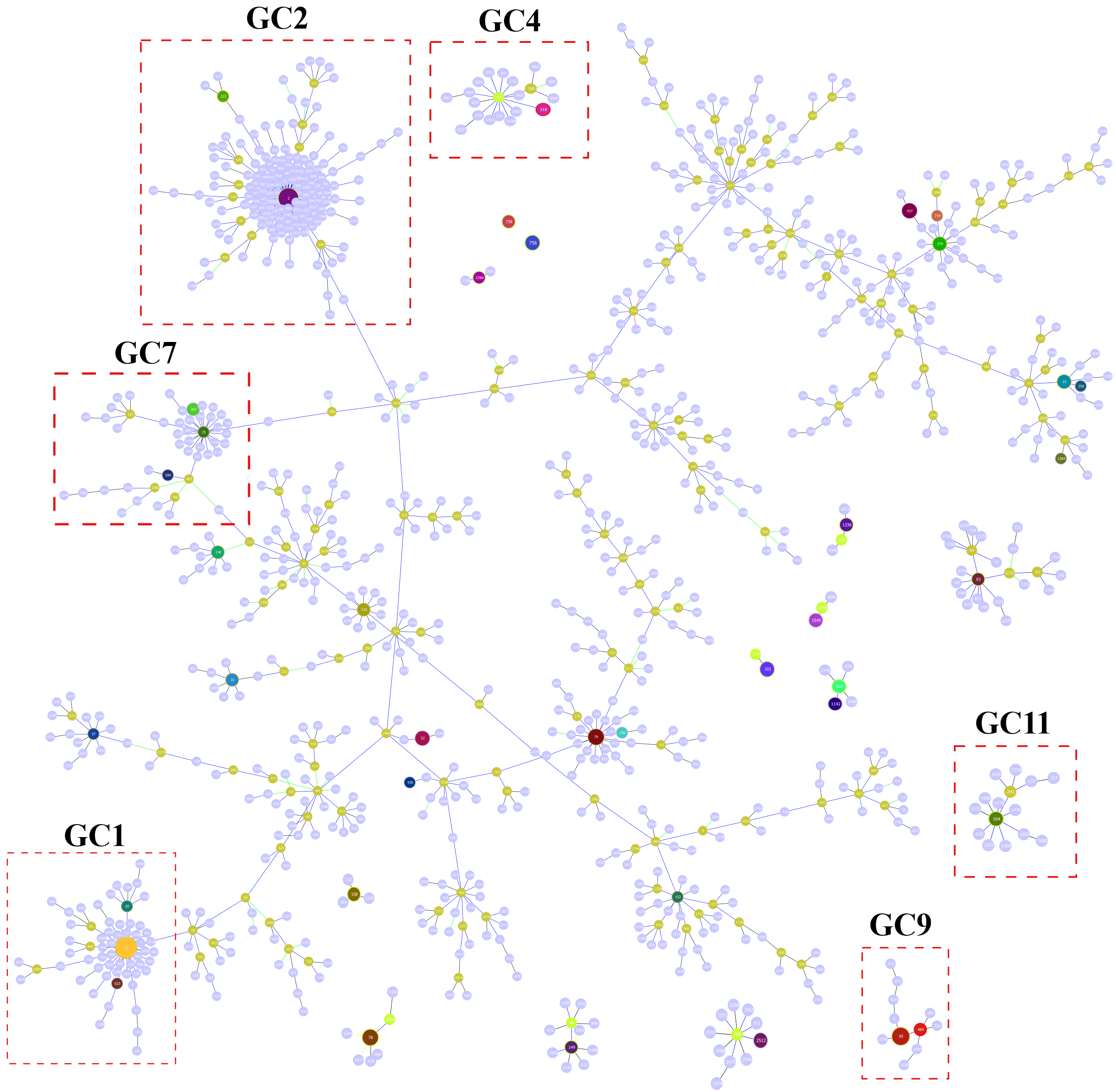
Supplementary Figure 3: Minimum Spanning Tree diagram of the STs of the zot-positive A. baumannii strains retrieved from the NCBI database and the strains collected in the current study, together with all Pasteur STs deposited in the PubMLST database.** STs are denoted by the numbers inside the circles. Light blue and light green circles correspond to STs in the database and clonal complexes, respectively. Circles of other colors correspond to the STs to which the zot-positive strains belong.

**Supplementary Figure 4: Similarity matrix of Zot proteins encoded by *A. baumannii* from the current study and Zot proteins encoded by other bacterial pathogens.**

**Supplementary Figure 5: MSA of Zot proteins encoded by *A. baumannii* from the current study and Zot proteins encoded by other bacterial pathogens.** Walker A, Walker B, and Zot receptor binding sites are highlighted by red squares.

**
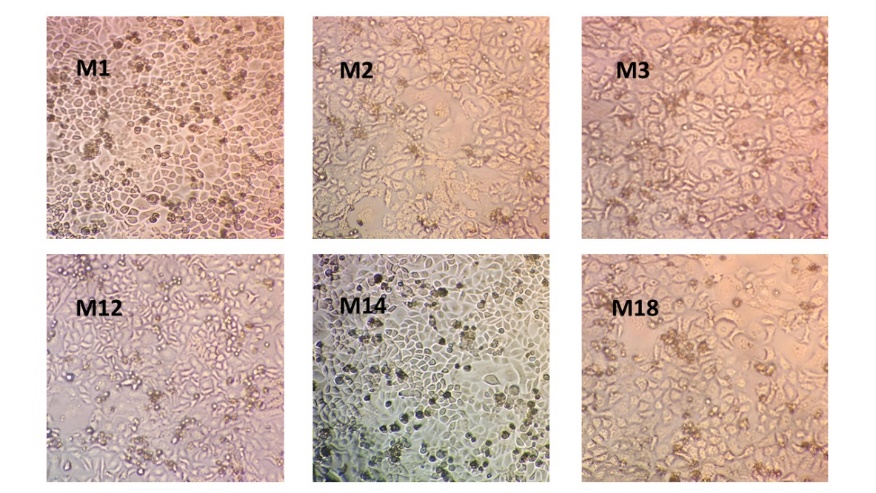
**

**Supplementary Figure 6: Morphological changes in Caco-2 cell line after incubation with CFSs for 48 hours.** The images were captured using an inverted light microscope (magnification 100X). M2, M3, M12, and M18 are *zot*-positive strains, while M14 carries an incomplete *zot*-coding gene. M1 represents the negative control (*zot*-negative strain).

**References:**

1. Narancic, J., D. Gavric, R. Kostanjsek, and P. Knezevic (2024) First Characterization of Acinetobacter baumannii-Specific Filamentous Phages. Viruses 16(6) 857.
